# Kallistatin protects against sepsis-related acute lung injury via inhibiting inflammation and apoptosis

**DOI:** 10.1038/srep12463

**Published:** 2015-07-22

**Authors:** Wei-Chieh Lin, Chang-Wen Chen, Yu-Wen Huang, Lee Chao, Julie Chao, Yee-Shin Lin, Chiou-Feng Lin

**Affiliations:** 1 Department of Internal Medicine, National Cheng Kung University Hospital, College of Medicine, National Cheng Kung University, Tainan, Taiwan; 2 Department of Biochemistry and Molecular Biology, Medical University of South Carolina, Charleston, South Carolina, USA; 3 Department of Microbiology and Immunology, College of Medicine, National Cheng Kung University, Tainan, Taiwan; 4 Center of Infectious Disease and Signaling Research, National Cheng Kung University, Tainan, Taiwan; 5 Graduate Institute of Medical Sciences, College of Medicine, Taipei Medical University, Taipei, Taiwan; 6 Department of Microbiology and Immunology, College of Medicine, Taipei Medical University, Taipei, Taiwan

## Abstract

Kallistatin, an endogenous plasma protein, exhibits pleiotropic properties in inhibiting inflammation, oxidative stress and apoptosis, as evidenced in various animal models and cultured cells. Here, we demonstrate that kallistatin levels were positively correlated with the concentration of total protein in bronchoalveolar lavage fluids (BALF) from patients with sepsis-related acute respiratory distress syndrome (ARDS), indicating a compensatory mechanism. Lower ratio of kallistatin to total protein in BALF showed a significant trend toward elevated neutrophil counts (P = 0.002) in BALF and increased mortality (P = 0.046). In lipopolysaccharide (LPS)-treated mice, expression of human kallistatin in lung by gene transfer with human kallistatin-encoding plasmid ameliorated acute lung injury (ALI) and reduced cytokine/chemokine levels in BALF. These mice exhibited attenuated lung epithelial apoptosis and decreased Fas/FasL expression compared to the control mice. Mouse survival was improved by kallistatin gene transfer or recombinant human kallistatin treatment after LPS challenge. In LPS-stimulated A549 human lung epithelial cells, kallistatin attenuated apoptosis, down-regulated Fas/FasL signaling, suppressed intracellular reactive oxygen species (ROS) and inhibited ROS-mediated NF-κB activation and inflammation. Furthermore, LPS-induced apoptosis was blocked by antioxidant N-acetylcysteine or NF-κB inhibitor via down-regulating Fas expression. These findings suggest the therapeutic potential of kallistatin for sepsis-related ALI/ARDS.

Acute lung injury (ALI) and its more severe form, acute respiratory distress syndrome (ARDS), are characterized by diffuse lung inflammation and alveolar-capillary destruction, resulting in alveolar flooding and acute respiratory failure. Although mortality from ALI has decreased in the last decade due to the implementation of lung-protective ventilation strategies^1^, prone position^2^ and fluid-conservative therapy^3^, ALI-related lethality remains high (30–40%)^4^. The pathogenesis of ALI is generally believed to be caused by lung inflammation and cell apoptosis, characterized by the accumulation of inflammatory cells; the aberrant release of proteases, reactive oxygen species (ROS), and proinflammatory cytokines; and a sustained loss of normal alveolar capillary barrier function^5^. A variety of pharmacologic therapies have been evaluated, such as glucocorticoids, surfactants, inhaled nitric oxide, antioxidants, protease inhibitors, and anti-inflammatory, antithrombotic and fibrinolytic treatments, but none of them has proven to be effective^6^,^7^,^8^.

Kallistatin is a plasma protein and tissue kallikrein-binding protein^9^. Independent of its interaction with tissue kallikrein, kallistatin has been shown to exert pleiotropic functions, such as anti-angiogenesis, anti-apoptosis, anti-inflammation, and anti-oxidative stress^10^,^11^,^12^,^13^,^14^,^15^. Kallistatin levels in plasma are significantly reduced in patients with liver disease, and correlate with sepsis and disease severity in patients with community-acquired pneumonia^9^,^16^. Transgenic mice overexpressing kallistatin exhibit enhanced resistance to lipopolysaccharide (LPS)-induced lethality^17^. This protective effect is also observed in group A streptococcus-infected mice with kallistatin gene transfer, and in polymicrobial septic mice treated with recombinant kallistatin^18^,^19^. Furthermore, kallistatin inhibits inflammatory cell infiltration and oxidative stress in animal models of myocardial ischemia-reperfusion injury, chronic myocardial infarction and salt-induced renal injury^15^,^20^,^21^. In the carbon tetrachloride-induced liver injury mouse model, transgenic expression of kallistatin attenuates liver damage through reduction of oxidative stress^22^. Moreover, depletion of endogenous kallistatin by antibody injection aggravates organ damage, inflammation and oxidative stress in hypertensive rats^23^. Taken together, these findings indicate that kallistatin exerts protective functions via various biological actions. However, the role of kallistatin in ALI has not yet been described.

In the present study, we provide evidence that kallistatin protects against ALI. Our results show that levels of kallistatin in bronchoalveolar lavage fluids (BALF) were associated with the lung inflammation and outcome in patients with sepsis-related ARDS. Kallistatin gene delivery or kallistatin protein administration significantly ameliorated ALI in LPS-treated mice. Moreover, in human lung epithelial cells, kallistatin attenuated LPS-induced inflammation and apoptosis by inhibiting ROS generation and NF-κB activation, subsequently down-regulating Fas/FasL signaling.

## Results

### BALF levels of kallistatin are associated with the lung inflammation and outcome in patients with sepsis-related ARDS

**Figure 1:**
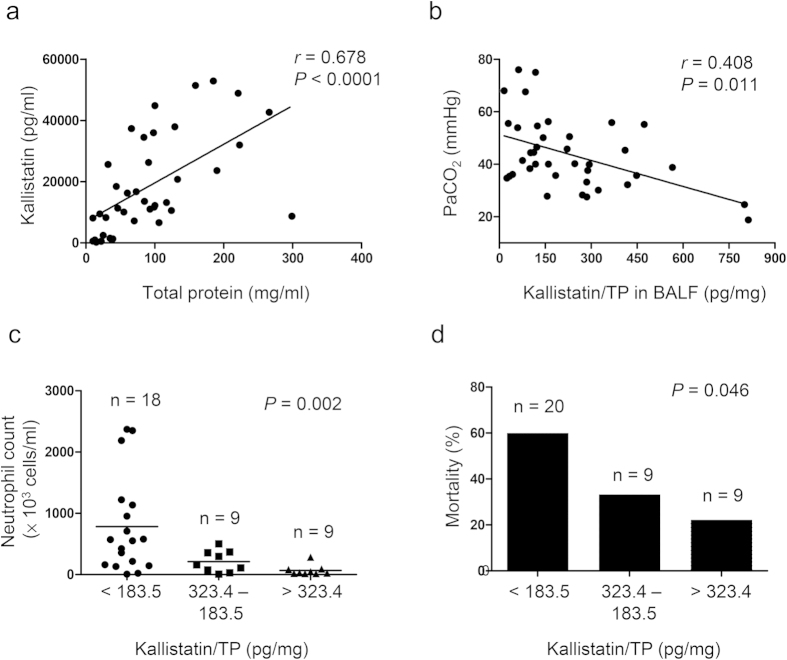
Effect of kallistatin on the lung inflammation and outcome of ARDS patients. BALF samples were collected within 24 h once the diagnosis of ARDS was established. Kallistatin levels were measured using ELISA. (**a**) Kallistatin levels were positively correlated with total protein (TP) in BALF. (**b**) Correlation between kallistatin/TP ratio and PaCO_2_ value is shown. Spearman correlation-of-rank coefficient was used to analyze the association between all studied parameters. Neutrophil counts in BALF (**c**) and observed mortality (**d**) according to three categories of kallistatin/TP ratio. Test for trend across categories was performed.

Our previous studies have demonstrated that plasma kallistatin levels may predict the outcomes of patients with severe community-acquired pneumonia^16^. Herein, we tested whether BALF kallistatin levels are relevant to disease severity and mortality in patients with sepsis-related ARDS. A total of thirty-eight patients were included in the study. The causes of ARDS were primary pneumonia (n = 27) and sepsis of extrapulmonary origin (n = 11). The hospital mortality was 44.7% (Supplementary Table S1). We found that BALF kallistatin levels were positively correlated with the concentration of total protein in BALF (*r* = 0.678, P < 0.0001) (Fig. 1a). This result implicates that kallistatin could be secreted into alveoli from circulation as BALF total proteins reflect the extent of impaired alveolar-capillary barrier that leads to influx of protein. We thus normalized the BALF kallistatin into the kallistatin/total protein ratio, indicating the alveolar kallistatin levels under the same alveolar-capillary barrier conditions, to determine its effects on lung inflammation and patients’ outcomes. We demonstrated that the BALF kallistatin/total protein ratio was negatively correlated with the arterial partial pressure of carbon dioxide (PaCO_2_) levels (*r* = 0.408, P = 0.011) (Fig. 1b). A decrease in PaCO_2_ has been demonstrated to be more predictive of improved outcome than indices of oxygenation in ARDS patients treated with prone positioning^24^, which appears to be the same for patients with higher BALF kallistatin levels. We found a categoric decrease in BALF neutrophil counts with a significant trend test across three categories of kallistatin/total protein ratio (P = 0.002) (Fig. 1c). Moreover, in comparison with the overall mortality of 44.7%, a categoric decrease in death was observed from 60% in the lowest to 22.2% in the highest kallistatin/total protein ratio, with a significant test for trend across three categories (P = 0.046) (Fig. 1d). Our findings suggest that kallistatin in BALF is associated with the lung inflammation and outcome in patients with sepsis-related ARDS.

### Kallistatin gene or protein administration improves survival in mice with LPS-induced ALI

**Figure 2:**
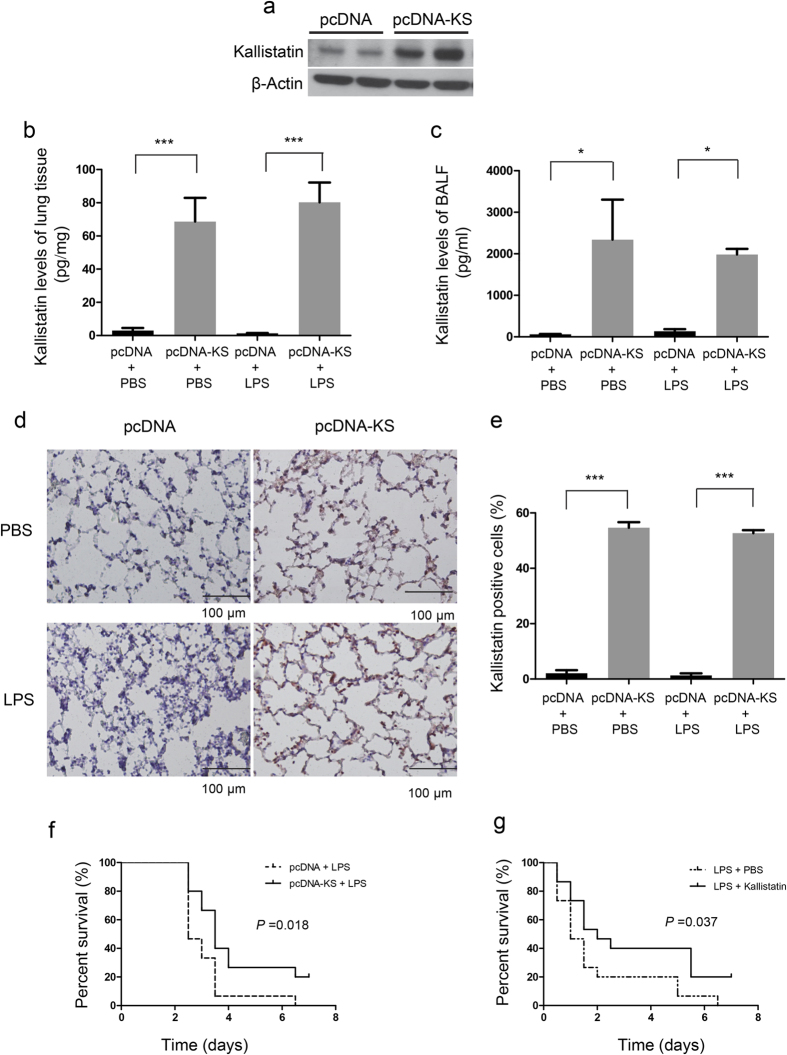
Kallistatin gene or protein delivery improves survival in mice with LPS-induced lung injury. Plasmid DNA carrying human kallistatin gene transfected mouse lungs via intranasal route. (**a**) Lung tissues were collected 16 h after mice received plasmid DNA carrying human kallistatin gene (pcDNA-KS) or control plasmid DNA (pcDNA), and the protein levels of kallistatin were evaluated by western blotting. (**b**,**c**) After 16 h of plasmid DNA transfer, mice were treated either with LPS (50 μl, 10 mg/ml) or with PBS intranasally for another 24 h (n = 5 per group). Thereafter, lung tissues and BALF were obtained, and kallistatin levels were measured using ELISA. Data are shown as the mean ± SEM and are representative of three independent experiments. *P < 0.05 and ***P < 0.001. (**d**) The lung sections were also stained with human kallistatin antibody and examined by immunohistochemistry. *Brown* staining indicates kallistatin expression. Representative images of lung section are presented. Scale bars, 100 μm. (**e**) The percentages of kallistatin-positive cells were counted and data were obtained from three independent experiments. Data are presented as mean ± SEM. ***P < 0.001. All data were analyzed by one-way ANOVA with Bonferroni’s *post hoc* test. Survival rates were observed for 7 days. (**f**) Survival curve of mice (n = 15, each group) with plasmid DNA expressing human kallistatin (pcDNA-KS) or control plasmid DNA (pcDNA) 16 h before administration of two doses of LPS (50 μl, 10 mg/ml) 24 h apart. (**g**) Survival curve of mice (n = 15, each group) treated with 50 μl recombinant human kallistatin (50 μg/ml) or PBS via intranasal route 6 h after each LPS challenge. The survival curves were compared using the log-rank test.

To further determine the protective effects of kallistatin on sepsis-related ALI, we transfected mice with plasmid DNA carrying human kallistatin cDNA (pcDNA3.1-KS) or control plasmid (pcDNA3.1) 10 μg diluted in 50 μl of phosphate buffered saline (PBS) by intranasal administration 16 h before ALI induced by LPS. Intranasal plasmid DNA delivery for the treatment of lung diseases has been widely used in previous studies^25^. Intranasal administration enables drug delivery to target cells in the lungs and serves as an effective, noninvasive and safe approach^25^. LPS is a component of the outer membrane of gram-negative bacteria and is well-known as a common cause of sepsis. Therefore, LPS administration has been widely used as a model of sepsis-related ALI^26^. In present study, we treated mice with high dose of LPS (50 μl, 10 mg/ml) intranasally to create a lethal lung injury model to imitate clinical presentation of ARDS patients. The dose of LPS was chosen based on the other and our previous studies^27^,^28^,^29^. At 24 h after LPS challenge, expression of human kallistatin was detected in BALF and lung tissue of mice receiving kallistatin gene delivery, but not in mice injected with control plasmid DNA in the absence or presence of LPS challenge (Fig. 2a–c). There were no statistically significant differences in BALF and lung tissue levels of kallistatin between kallistatin gene transfer with and without LPS challenge (Fig. 2b,c). Immunohistochemical staining shows that kallistatin was expressed in more than 50 percent of lung epithelial cells (Fig. 2d,e). In survival experiments, mice received two doses of LPS (50 μl; 10 mg/ml) 24 h apart to reach a lethal dose according to our previous study^29^. Such dosage of LPS can cause over 50% reduced survival rate after 4 days. The survival rate was assessed for 7 days. As shown in Fig. 2f, all mice treated with control plasmid died within 7 days, and mice expressing kallistatin had a 20% survival rate. The therapeutic potential of kallistatin was verified in mice treated with human kallistatin protein (50 μl; 50 μg/ml) twice via intranasal route 6 h after each LPS exposure (Fig. 2g). Results showed that exogenous administration of kallistatin improved the survival rate of mice by 20% when challenged with LPS. We conclude that kallistatin provides survival benefits in LPS-induced ALI.

### Kallistatin ameliorates lung injury and inflammation in LPS-challenged mice and A549 cells

**Figure 3:**
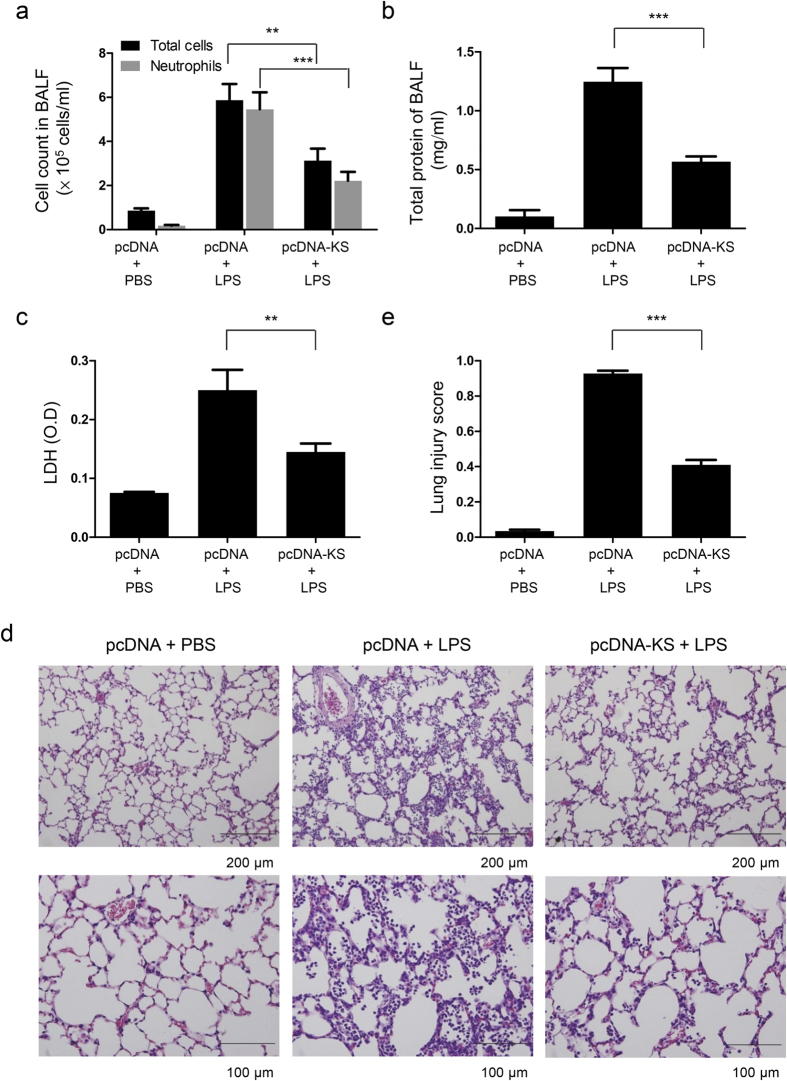
Kallistatin gene delivery significantly attenuates LPS-induced lung injury. Mice receiving plasmid DNA expressing human kallistatin (pcDNA-KS) or control plasmid DNA (pcDNA) were killed 24 h after LPS (50 μl, 10 mg/ml intranasally) or PBS treatment. (**a**) Total BALF cells and neutrophils were quantified by cytospin and hemocytometer. Lung injury severity was determined by measuring (**b**) total protein levels and (**c**) LDH activity in BALF. For each treatment a mean value of at least n = 7 per group, from three separate experiments, are shown. (**d**) Representative images of hematoxylin and eosin–stained lung sections showed attenuated alveolar cell infiltrates and alveolar interstitial thickening in mice receiving kallistatin gene transfer compared with control mice upon LPS challenge. Scale bars, 200 μm (top panel), 100 μm (bottom panel). (**e**) The lung injury scores were ass**e**ssed in 20 fields (×400 magnification) of lung sections, taken from 7 mice for each group. All data are shown as the mean ± SEM and were analyzed by one-way ANOVA with Bonferroni’s *post hoc* test. **P < 0.01 and ***P < 0.001.

**Figure 4:**
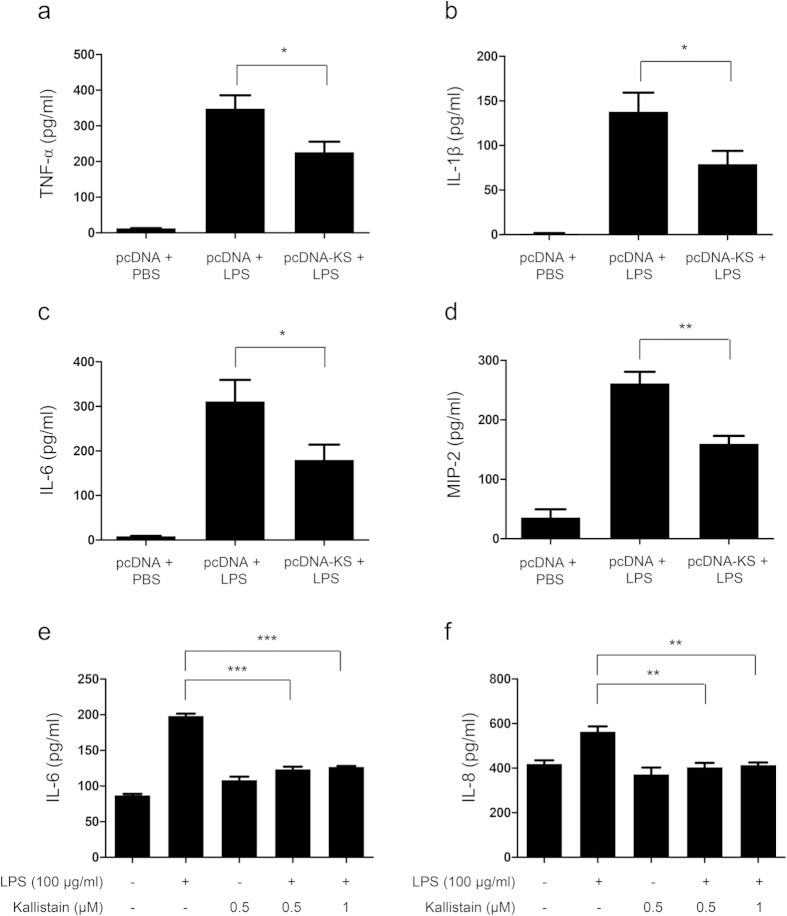
Kallistatin gene delivery or kallistatin protein treatment significantly decreases cytokine/chemokine levels in BALF and in A549 cultured medium. Mice receiving plasmid DNA encoding human kallistatin (pcDNA-KS) or control plasmid DNA (pcDNA) were killed 24 h after LPS (50 μl, 10 mg/ml intranasally) or PBS administration. Levels of (**a**) TNF-α, (**b**) IL-1β, (**c**) IL-6, and (**d**) MIP-2 were measured in BALF **b**y ELISA. For each treatment a mean value of at least n = 6 per group, from three separate experiments, are shown. Data are shown as mean ± SEM. A549 cells were pretreated with recombinant human kallistatin at indicated doses for 1 h and then exposed to LPS (100 μg/ml) for another 24 h. Levels of (**e**) IL-6 and (**f**) IL-8 were measured in cultured medium by ELISA. The data are shown as the mean ± SEM of triplicate cultures and are representative of three independent experiments. All data were analyzed by one-way ANOVA with Bonferroni’s *post hoc* test. *P < 0.05, **P < 0.01, and ***P < 0.001.

Next, we examined the severity of lung injury and inflammation in mice when challenged with LPS. Total protein, total cell and neutrophil counts, and lactate dehydrogenase (LDH) activity in BALF were attenuated by kallistatin gene transfer upon LPS challenge (Fig. 3a–c). Histotological analysis showed that mice receiving kallistatin gene transfer had substantially less inflammatory cell infiltration, edema, hemorrhage, and thickness of alveolar walls, and decreased lung injury scores, in comparison to control mice treated with LPS (Fig. 3d,e). Numerous inflammatory mediators have been identified to contribute to the pathogenesis of ALI. Therefore, we determined the expression of several cytokines and chemokines in mice with LPS-induced ALI. Our results showed that the levels of tumor necrosis factor (TNF)-α, interleukin (IL)-1β, IL-6, and macrophage inflammatory protein (MIP)-2 in BALF were markedly decreased in mice receiving kallistatin gene transfer compared to control mice 24 h after LPS challenge (Fig. 4a–d). To further verify our findings, we examined the effect of kallistatin on A549 cells, a human lung epithelial cell line, by administrating human kallistatin protein 1 h before LPS exposure. At 24 h after LPS application, kallistatin significantly lowered IL-6 and IL-8 levels compared to LPS treatment alone (Fig. 4e,f). Taken together, our data suggest that kallistatin ameliorates LPS-induced lung injury and inflammation.

### Kallistatin protects the lung against LPS-induced apoptosis through down-regulation of Fas/FasL signaling

**Figure 5:**
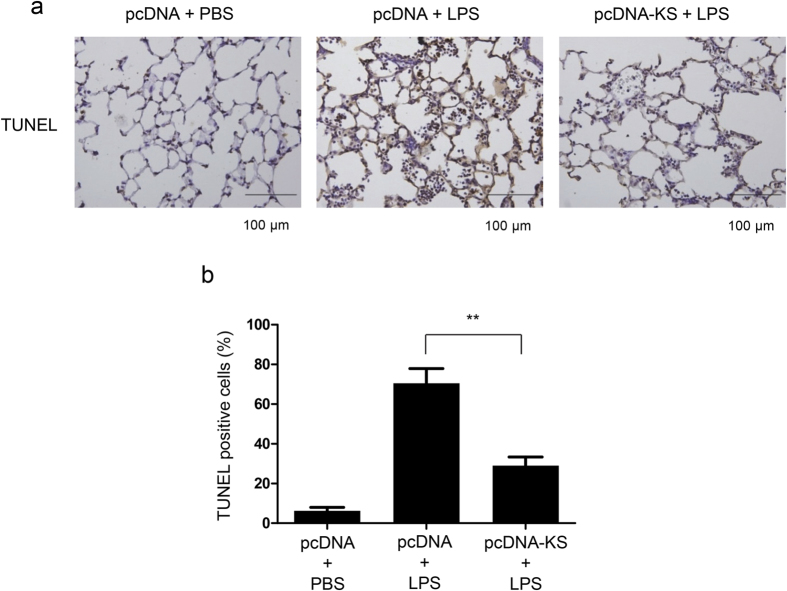
Kallistatin gene transfer attenuates lung epithelial apoptosis in lung tissues. Mice receiving plasmid DNA encoding human kallistatin (pcDNA-KS) or control plasmid DNA (pcDNA) were killed 24 h after either LPS (50 μl, 10 mg/ml intranasally) or PBS administration. (**a**) Representative images of TUNEL–stained lung sections are shown. Results are representative of three independent experiments. *Brown* staining indicates TUNEL-positive cells. Scale bars, 100 μm. (**b**) The percentages of apoptotic lung epithelial cells were counted and data were obtained from three independent experiments. Data are presented as mean ± SEM and were analyzed by one-way ANOVA with Bonferroni’s *post hoc* test. **P < 0.01.

**Figure 6:**
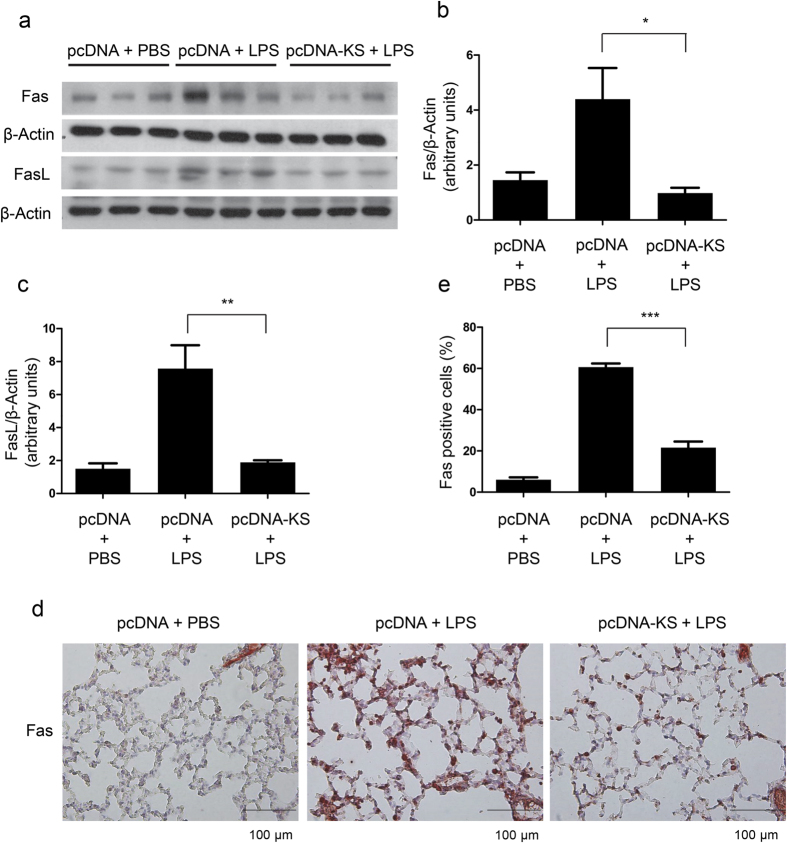
Kallistatin gene transfer attenuates LPS-induced elevation of Fas/FasL expression in lung tissues. Mice were treated with either plasmid DNA expressing human kallistatin (pcDNA-KS) or control plasmid DNA (pcDNA). After 16 h, mice were treated either with LPS (50 μl, 10 mg/ml) or with PBS intranasally for another 24 h. (**a**) Lung lysates underwent western blotting for Fas and FasL. Representative images are shown from three independent experiments. (**b**,**c**) Densitometric analysis was performed using Image J software and shown as fold change from pcDNA+PBS. Data are shown as the mean ± SEM; n = 3 per group. *P < 0.05, **P < 0.01. (**d**) Representative images of Fas-stained lung sections by immunohistochemistry. *Red* staining indicates Fas-positive lung epithelial cells. Scale bars, 100 μm. (**e**) The percentages of Fas-positive lung epithelial cells were counted and data were obtained from three independent experiments. Data are presented as mean ± SEM. ***P < 0.001. All data were analyzed by one-way ANOVA with Bonferroni’s *post hoc* test.

**Figure 7:**
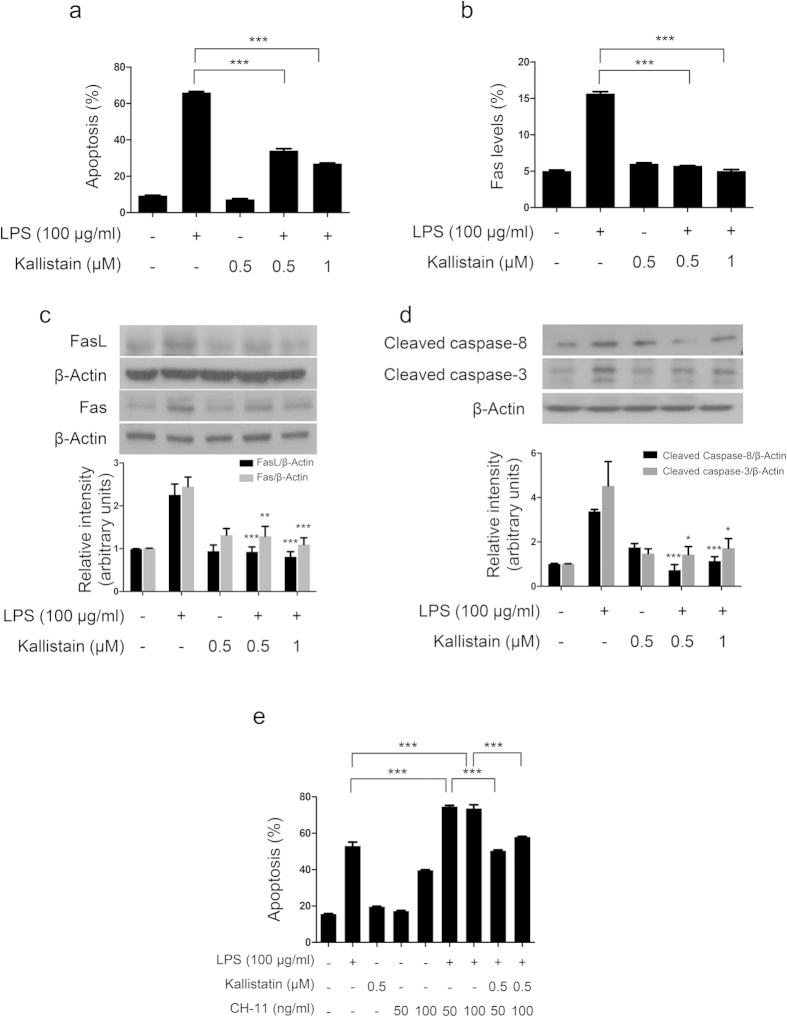
Kallistatin treatment attenuates LPS-induced apoptosis in A549 cells through down-regulation of Fas/FasL signaling. A549 cells were pretreated with recombinant human kallistatin at indicated doses for 1 h and then exposed to LPS (100 μg/ml) for another 48 h. (**a**) Apoptosis was detected using PI staining followed by flow cytometry. The data are presented as the percentage of apoptotic cells. (**b**) Fas levels were determined by immunostaining followed by flow cytometry, and the results are shown as the averaged percentages of Fas-positive cells. (**c**,**d**) Western blot analysis was used to determine the protein levels of Fas and FasL and cleaved caspase-8 and -3; β-actin was used as an internal control. Histogram indicates the relative band intensity of western blot from three independent experiments. Data are shown as the mean ± SEM. *P < 0.05, **P < 0.01, and ***P < 0.001 compared to LPS-treated alone group. (**e**) A549 cells were pretreated with recombinant human kallistatin (0.5 μM) for 1 h and then exposed to LPS (100 μg/ml) and Fas-activating antibody CH-11 at indicated doses for another 48 h. PI staining and flow cytometry were used to determine cell apoptosis. The results shown are representative of three independent experiments. Data are shown as the mean ± SEM of triplicate cultures. ***P < 0.001. All data were analyzed by one-way ANOVA with Bonferroni’s *post hoc* test.

Lung epithelial cell apoptosis was increased in mice upon LPS challenge, as determined by terminal deoxynucleotidyl transferase (TdT)-mediated dUTP–biotin nick-end labeling (TUNEL) staining, whereas kallistatin gene delivery attenuated LPS-induced cell death (Fig. 5a). Quantitation of TUNEL staining shows that kallistatin gene transfer significantly attenuated the increase in apoptotic lung epithelial cells after LPS challenge (Fig. 5b). Cleaved caspase-3 levels were also found to be elevated in lung epithelial cells after LPS stimulation, but were suppressed in mice with kallistatin gene delivery (Supplementary Figure S1). The Fas/FasL pathway has previously been demonstrated to contribute to lung epithelial cell apoptosis in LPS-induced ALI^30^. Therefore, we examined the effect of kallistatin on the regulation of Fas/FasL expression. As shown in Fig. 6a–c, human kallistatin gene transfer down-regulated LPS-induced Fas/FasL expression. Immunohistochemical staining demonstrates that kallistatin gene transfer reduced the elevated Fas expression of lung epithelial cells in response to LPS challenge (Fig. 6d,e). To confirm our *in vivo* findings *in vitro*, we pretreated A549 cells for 1 h with human kallistatin, then exposed cells to LPS for another 48 h and assessed for apoptosis, Fas/FasL expression, and caspase-3 and -8 activation. The results showed that kallistatin attenuated LPS-induced cell apoptosis (Fig. 7a), antagonized Fas/FasL expression (Fig. 7b,c), and suppressed caspase-3 and -8 activation (Fig. 7d and Supplementary Figure S2). To further verify the role of Fas in mediating LPS-induced A549 apoptosis, cells were co-treated with LPS and CH-11, a human Fas-activating antibody, for 48 h. The results revealed that CH-11 enhanced LPS-induced apoptosis, and its effect was attenuated by kallistatin pretreatment 1 h before LPS and CH-11 exposure (Fig. 7e). To support the mechanism of Fas contributing to LPS-induced apoptosis, ZB4, a Fas-blocking antibody, was administered 1 h before LPS treatment for 48 h. The result showed that ZB4 reduced LPS-induced apoptosis (Supplementary Figure S3). Taken together, these findings indicate that kallistatin protects the lung against LPS-induced apoptosis through down-regulation of Fas/FasL signaling.

### Kallistatin decreases LPS-induced ROS generation and NF-κB activation in A549 cells

**Figure 8:**
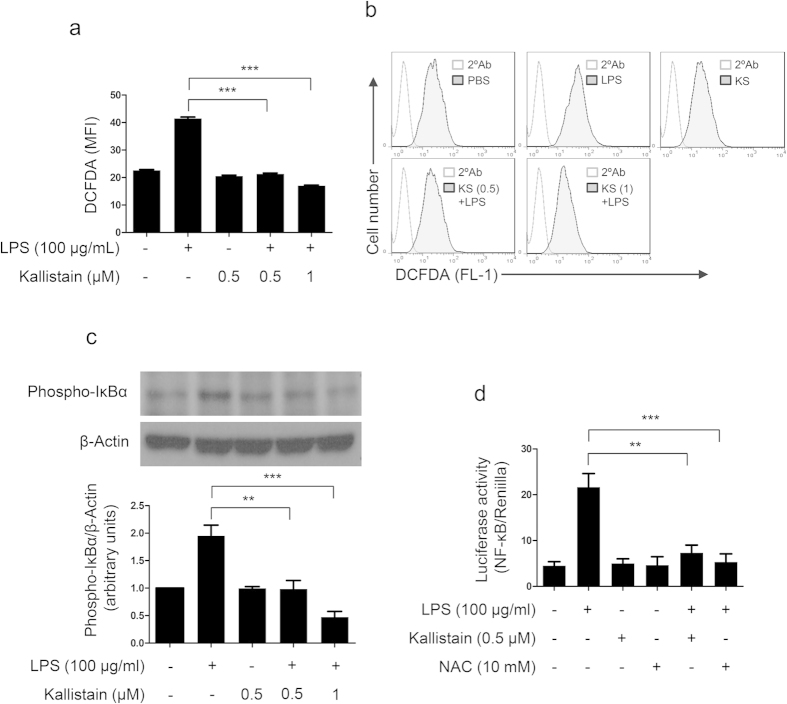
Kallistatin treatment reduces LPS-induced ROS generation and inhibits L*P*S-induced NF-κB activation in A549 cells. Cells were exposed to LPS (100 μg/ml) or pretreated with recombinant human kallistatin at indicated doses for 1 h and then exposed to LPS for another 24 h. (**a**) Intracellular ROS levels were measured with CM-H_2_DCFDA staining followed by flow cytometric analysis. Data are presented as the averaged median fluorescence intensity (MFI) **a**nd shown as the mean ± SEM. ***P < 0.001. (**b**) Representative histograms obtained from the flow cytometric analysis showing the generation of fluorescent oxidized DCF in A549 cells. Data are representative of three independent experiments. (**c**) Western blot analysis was used to determine the levels of phospho-IκBα; β-actin was used as an internal control. Results are representative of three independent experiments. Histogram indicates the relative band intensity of western blot from three independent experiments. Data are shown as the mean ± SEM. **P < 0.01 and ***P < 0.001. (**d**) Luciferase reporter assays were used to determine the transactivation ratio of NF-κB to *Renilla* in the A549 cells treated with LPS for 24 h with or without a 1 h pretreatment of kallistatin (0.5 μM) or antioxidant NAC (10 mM). Data are shown as the mean ± SEM of triplicate cultures and are representative of three independent experiments. **P < 0.01 and ***P < 0.001. All data were analyzed by one-way ANOVA with Bonferroni’s *post hoc* test.

Given previous evidence of ROS in mediating cell death^31^,^32^ and the antioxidant properties of kallistatin^13^,^21^,^33^, we investigated the effect of kallistatin on LPS-induced ROS generation in A549 cells. As shown in Fig. 8a,b, kallistatin significantly inhibited LPS-induced ROS generation. ROS has been reported to be associated with NF-κB activation during LPS/Toll-like receptor 4 (TLR4) signaling, and are implicated in a variety of cellular functions, including inflammation and cell death^34^,^35^. Therefore, we next examined the effect of kallistatin or N-acetylcysteine (NAC), a strong antioxidant, on LPS-induced NF-κB activation. LPS caused significant activation of NF-κB, whereas pretreatment of A549 cells with kallistatin or NAC for 1 h before LPS exposure dramatically inhibited phospho-IκB and NF-κB activation (Fig. 8c,d). Also, we found that kallistatin attenuated LPS-induced TLR4 expression (Supplementary Figure S4). Taken together, these results indicate that kallistatin inhibits NF-κB activation in response to LPS through prevention of TLR4-mediated ROS generation.

### Kallistatin, NAC, and NF-κB inhibitor block LPS-induced Fas expression and apoptosis

**Figure 9:**
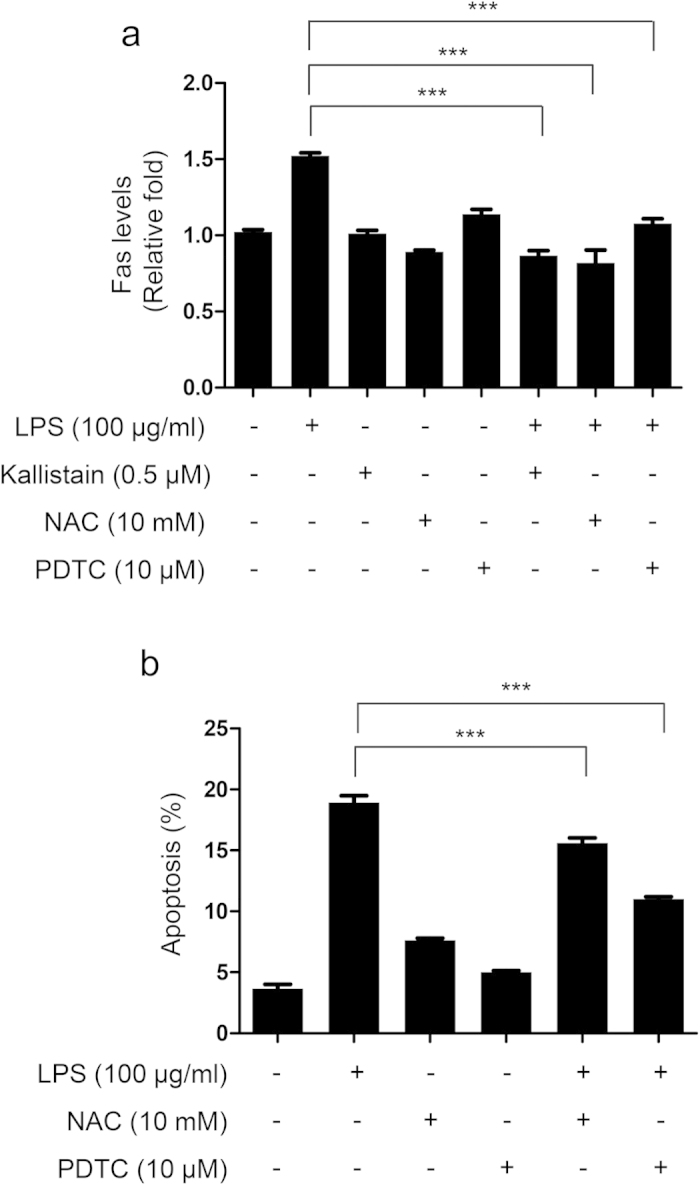
Inhibition of ROS and NF-κB attenuates LPS-induced Fas expression and cell death in A549 cells. Cells were pretreated with or without recombinant human kallistatin (0.5 μM), NAC (10 mM) or NF-κB inhibitor PDTC (10 μM) for 1 h and then exposed to LPS (100 μg/ml) for 24 h. (**a**) Fas levels were measured using immunostaining followed by flow cytometry. Data are presented as fold change when normalized with the control. (**b**) PI staining and flow cytometric analysis were used to detect apoptosis in the presence or absence of NAC (10 mM) or NF-κB inhibitor PDTC (10 μM) 1 h before LPS treatment for 48 h. The data are shown as the mean ± SEM; experiments were performed in three independent experiments. ***P < 0.001. All data were analyzed by one-way ANOVA with Bonferroni’s *post hoc* test.

Based on our observations that LPS induces cell death, generates ROS, and activates NF-κB in A549 cells, we hypothesized that both ROS and NF-κB may, in turn, mediate LPS-induced Fas expression and apoptosis. We therefore used pyrrolidine dithiocarbamate (PDTC), a specific inhibitor of NF-κB, to evaluate whether NF-κB activation is required for LPS-induced Fas expression. In addition, NAC was administered to verify that ROS generation is essential for activation of LPS-induced NF-κB/Fas signaling. Thus, A549 cells were pretreated with kallistatin, NAC or PDTC for 1 h before LPS exposure, and Fas expression was detected using flow cytometry. As seen in Fig. 9a,b, cells pretreated with kallistatin, NAC or PDTC attenuated LPS-induced Fas expression and apoptosis. These findings indicate that kallistatin prevents LPS-induced cell apoptosis and Fas expression by inhibiting oxidative stress and NF-κB activation.

## Discussion

In the present study, we show that reduced kallistatin levels are strongly associated with increased lung inflammation and mortality of sepsis-related ARDS in hospitalized patients. These observations were supported by an *in vivo* study of a mouse model of LPS-induced ALI, and *in vitro* with LPS-stimulated lung epithelial cells. It has been reported that intranasal plasmid DNA delivery is biologically effective and a noninvasive, safe and lung-targeted approach without the need for viral vectors or transfection agents, thereby obviating potential concerns for the specific immune response and toxicity^25^. Using the technique of kallistatin gene transfer, we found that kallistatin alleviated LPS-induced lung injury, inflammation and lung epithelial cell apoptosis, and down-regulated Fas/FasL expression. Moreover, both kallistatin gene transfer and recombinant kallistatin administration improved the survival of mice upon LPS challenge. In LPS-stimulated A549 cells, kallistatin was shown to reduce ROS production, inhibit NF-κB activation, and attenuate Fas-mediated cell apoptosis. In addition, we found that the LPS-induced cell apoptosis was attenuated by administration of an antioxidant and NF-κB inhibitor through down-regulation of Fas/FasL signaling, indicating that the protective role of kallistatin in LPS-induced ALI and lung epithelial cell apoptosis is mediated by decreased ROS generation and NF-κB activation, subsequently down-regulating Fas/FasL signaling.

Sepsis is the major cause of ALI development in the clinical setting^6^. Our recent studies have shown that lower plasma levels of kallistatin have a trend toward predicting worse clinical outcomes in community-acquired pneumonia^16^, and kallistatin provides protection against organ damage and survival benefit in group A streptococcus-infected mice^18^. Moreover, previous studies have demonstrated that plasma kallistatin levels are significantly reduced in patients with septic shock^9^, and transgenic mice expressing kallistatin are more resistant to LPS-induced mortality compared to control littermates^17^. ALI is characterized by intensive lung inflammation and alveolar damage, which can lead to multi-organ dysfunction and death. Our results showed that kallistatin levels were positively correlated with the concentration of total protein in BALF from ARDS patients, implicating kallistatin as a function of the extent of impaired alveolar-capillary barrier. It can be explained by the impaired alveolar-capillary barrier developed during ALI, leading to influx of protein, including kallistatin, from circulation. This pathophysiologic effect can also explain why plasma kallistatin levels are decreased in sepsis and pneumonia^9^,^16^. Accordingly, we suggest that BALF kallistatin levels rise in response to ALI development, as a compensatory mechanism, to mitigate the lung inflammation by its pleiotropic effects^21^. To support this observation, we further normalized kallistatin in BALF by alveolar protein leak as the kallistatin/total protein ratio to represent the alveolar kallistatin levels under the same alveolar-capillary barrier conditions. We found that the lower kallistatin/total protein ratio in BALF had a strong trend toward higher neutrophil counts in BALF and predicting worse clinical outcomes, as indicated by increased PaCO_2_ value and mortality. These observations were confirmed by our animal studies showing improved survival rate by kallistatin gene transfer or recombinant kallistatin administration after lethal dose of LPS challenge. In addition, we found that kallistatin gene transfer alleviated lung injury induced by LPS, as assessed by histology, decreased neutrophil counts, and reduced levels of total protein and LDH activity in BALF. These observations suggest a protective role of kallistatin in sepsis-related ALI.

Compelling evidence has shown that LPS induces ROS generation in lung epithelial cells^31^. ROS generated under physiological conditions maintain cell homeostasis, but their excessive generation can activate redox-sensitive transcription factors such as NF-κB, thereby provoking the cell inflammatory response and apoptosis^36^, thus contributing to the pathogenesis of ARDS. As shown in our *in vitro* study, we found a basal level of ROS generation in A549 cells over 24 h culture. The levels were significantly elevated following LPS treatment. Therefore, antioxidant strategies have been studied in order to attenuate LPS-induced lung injury^37^,^38^. Kallistatin has been shown to protect against salt-induced renal injury, inflammation, and fibrosis; carbon tetrachloride-induced liver fibrosis; and cardiac remodeling after chronic myocardial infarction via antioxidative stress and inhibition of NF-κB activation^20^,^21^,^33^. Likewise, our results showed that kallistatin suppressed LPS-induced ROS production and NF-κB activation in A549 cells. In agreement with previous studies reporting an association between TLR4 expression and ROS generation in various cell types^39^,^40^,^41^, we found that kallistatin also attenuated LPS-induced TLR4 expression. Moreover, we observed similar inhibitory effects of kallistatin on LPS-induced NF-κB activation in comparison with those of the antioxidant NAC. The anti-inflammatory effect of kallistatin was further indicated by decreased IL-6 and IL-8 levels in cultured medium of LPS-treated A549 cells, and lower levels of TNF-α, IL-1β and IL-6, and MIP-2 in BALF of mice subjected to LPS challenge. Our findings suggest that kallistatin reduces LPS-induced inflammation via suppressing ROS production and preventing redox-sensitive NF-κB activation and inflammatory signaling.

Accumulating evidence suggests that increased epithelial/endothelial cell apoptosis significantly contributes to the damage of the pulmonary alveolar-capillary barrier in ALI^5^. The Fas/FasL pathway has been demonstrated to play an essential role in the pathogenesis of lung epithelial apoptosis in sepsis or LPS-induced ALI^42^,^43^. The Fas receptor is a member of the tumor necrosis factor receptor family and is widely expressed on cell surfaces. This receptor can be activated by its ligand FasL or a cross-linking antibody (anti-Fas IgM) to invoke receptor oligomerization and apoptosis^44^. Activated Fas forms the death-inducing signaling complex (DISC), which is comprised of the Fas receptor, the adapter protein Fas-associated death domain protein (FADD), and multiple procaspase-8 molecules, leading to caspase-8 activation by autoprocessing. The initiator caspase-8 triggers a cascade that activates downstream effector caspases, such as caspase-3 and caspase-7^45^. In patients with ARDS, the levels of soluble Fas and FasL are increased in BALF at concentrations that induce apoptosis of distal lung epithelial cells^46^. Moreover, higher levels of soluble Fas and FasL in pulmonary edema fluid or BALF were associated with worse clinical outcomes in patients with ALI/ARDS^30^,^46^. In a mouse model of LPS-induced lung injury, up-regulation of Fas is accompanied by lung edema, neutrophil infiltration, and lung epithelial cell death^43^. Therefore, anti-apoptotic strategies targeting inhibition of the Fas/FasL pathway have been shown to improve survival in animal models of LPS-induced ALI and attenuate LPS-induced lung epithelial apoptosis^43^,^47^. In agreement with these findings, we found that LPS provoked lung epithelial apoptosis through the Fas/FasL pathway, although a minor activation of caspase and basal levels of Fas/FasL expression and cell death were observed in the control A549 cells in our long period of culture system. LPS-enhanced Fas/FasL expression was down-regulated by kallistatin, thereby suppressing caspase-8 and -3 activation, inhibiting apoptosis and attenuating lung injury. The anti-apoptotic effect of kallistatin is also observed in TNF-α-treated endothelial cells, in cardiomyocytes subjected to ischemia-reperfusion injury, and in the osteoarthritis rat model^13^,^14^,^15^. In contrast, a recent study showed that kallistatin induced apoptosis in the human colorectal cancer cells SW480 and HT-29 through activation of Fas/FasL pathway^48^. This contradictory result might be attributable to the differences in cell types and experimental models.

It has been reported that activation of Fas with an activating monoclonal antibody (CH-11) causes NF-κB activation and IL-8 secretion in human bronchiolar epithelial cells^49^. However, recent compelling studies indicate that NF-κB mediates apoptosis through Fas overexpression in various cell types^50^,^51^,^52^. It has been shown that NF-κB can recruit FADD and caspase-8 to the DISC to induce cell susceptibility to Fas-mediated apoptosis^50^. In agreement with these findings, our data also showed that kallistatin not only down-regulated the Fas/FasL pathway in association with apoptosis, but also decreased the production of ROS and the activation of NF-κB. Moreover, the expression of Fas/FasL induced by LPS was attenuated by pretreatment with kallistatin, antioxidant NAC and NF-κB inhibitor PDTC, indicating that kallistatin down-regulates Fas/FasL signaling in association with reduced ROS generation and NF-κB activation.

This is the first study to demonstrate that kallistatin protects against ALI induced by LPS through attenuation of oxidative stress and inactivation of NF-κB, thereby reducing inflammatory mediator production and down-regulating Fas-mediated apoptosis. These findings provide an insight into the role and mechanisms of kallistatin in LPS-induced ALI and offer a potential strategy in the prevention and treatment of ALI.

## Methods

### Patients

Patients who met the criteria of ARDS, as determined by the American College of Chest Physicians and Society of Critical Care Medicine Consensus Conference^53^, were enrolled at the medical ICU of the tertiary referral center of southern Taiwan. The ARDS of these patients was caused either by extrapulmonary sepsis or by pneumonia. Exclusion criteria were <16 years of age, had refractory respiratory failure (partial pressure of oxygen <60 mmHg with fraction of inspired oxygen 1.0), or had unstable hemodynamic status and lethal arrhythmia even under the use of a high-dose vasopressor and antiarrhythmia drug. All patients included in the study underwent fiberoptic bronchoscopy within 24 h once the diagnosis of ARDS was established. Written informed consent was obtained from all of the subjects or legal representatives if the patients were unconscious. A portion of BALF samples was collected afterwards for the study. The BALF from enrolled patients was centrifuged at 200 × *g* for 10 min at 4 °C to obtain the supernatant. The study was approved by the institutional review board of the National Cheng Kung University Hospital. The methods were carried out in accordance with the approved guidelines.

The baseline characteristics such as age and gender, laboratory data including PaO_2_/FiO_2_ ratio, and neutrophil counts and total protein levels in BALF were recorded on the first day of ARDS onset. The sequential organ failure assessment (SOFA) score^54^, acute physiology and chronic health evaluation II (APACHE II) score^55^ and lung injury score (LIS) as defined by Murray and colleagues^56^ were calculated in the first 24 h of ARDS onset.

### Mice

C3H/HeN breeder mice were obtained from The Jackson Laboratory (Bar Harbor, ME, USA). This strain of mouse was chosen based on our previous study as a model of LPS-induced lung inflammation and lethality in mice^29^. Their 6–8 wk-old progeny were used for experiments and maintained on standard laboratory food and water *ad libitum* in the Laboratory Animal Center of National Cheng Kung University. The animals were raised and cared for according to the guidelines of the Laboratory Animal Care and Use Committee of National Cheng Kung University. The experimental protocol was in accordance with the Animal Protection Law of Taiwan and was approved by the Institutional Animal Care and Use Committee at National Cheng Kung University for animal research.

### Reagents and antibodies

Recombinant human kallistatin and anti-kallistatin monoclonal antibodies were obtained from Drs. L. Chao and J. Chao. LPS (*Escherichia coli* O111:B6), N-acetylcysteine (NAC) and mouse monoclonal antibodies specific for β-actin and phospho-IκB were purchased from Sigma-Aldrich (St. Louis, MO, USA). Human Fas-activating antibody CH-11 and Fas-blocking antibody ZB4 were purchased from Millipore (Billerica, MA, USA). The NF-κB inhibitor pyrrolidine dithiocarbamate (PDTC) was obtained from Tocris Bioscience (Ellisville, MO, USA). Antibodies against Fas were obtained from BD Biosciences (San Jose, CA, USA) and Santa Cruz Biotechnology (Santa Cruz, CA, USA). Antibodies against FasL were obtained from R&D Systems (Minneapolis, MN, USA), and cleaved caspase-3 and -8 from Cell Signaling Technology (Danvers, MA, USA).

### Preparation of plasmid DNA and identification of human kallistatin in mouse lung

Plasmid DNA carrying human kallistatin gene pcDNA3.1-KS was prepared in the lab of Drs. L. Chao and J. Chao as previously described^18^, and amplified by *Escherichia coli* DH5α. The plasmids, including control and kallistatin, were prepared using a Qiagen EndoFree Plasmid Giga kit (Qiagen GmbH, Hilden, Germany) according to the manufacturer’s instructions. Control pcDNA3.1 or pcDNA3.1-KS (10 μg) was diluted in 50 μl of phosphate buffered saline (PBS) and given intranasally, under anaesthesia by intraperitoneal injection of 60 mg/kg of 2% sodium pentobarbital (Sigma-Aldrich), 16 h before the administration of LPS to mice. Expression of human kallistatin in mouse was analyzed by western blot using polyclonal antibody (R&D systems) and ELISA for detecting human kallistatin as previously described^9^.

### Purification and characterization of recombinant human kallistatin

His B-tagged pTrc expression vector carrying human kallistatin gene was transfected with human embryonic kidney cells (HEK293T) cells as established previously^19^,^57^. Transfected cell were then adapted into a serum-free suspension medium and the secrete proteins were concentrated by ammonium sulfate precipitation followed by nickel-affinity chromatography. Western blotting was carried out for verifying the purity and identity of human kallistatin by using a specific monoclonal antibody^9^,^19^

### Mouse model of LPS-induced ALI and assay of mouse lung injury

Before LPS or PBS treatment, all mice were anaesthetized with 60 mg/kg of 2% sodium pentobarbital intraperitoneally. Heart rate, chest wall movements and toe pinch were consistently monitored to detect the depth of anaesthesia. Lung injury was induced by intranasal instillation of 50 μl of LPS (10 mg/ml) as previously described^29^. Another group of mice underwent the same procedure but were treated with PBS. Animal samples were harvested 24 h after LPS administration under anaesthesia by an overdose of sodium pentobarbital. Bronchoalveolar lavage was obtained by cannulating the trachea with a 21-gauge catheter and then lavaging the lungs twice with 1 ml PBS. The recovered fluid was centrifuged and the supernatant was analyzed for protein concentration by Bio-Rad assay (Bio-Rad, Hercules, CA, USA). Cell pellets were pooled, resuspended in PBS, and the cell number was measured with a hemocytometer. The neutrophil content was determined by Giemsa staining. Lactate dehydrogenase (LDH) activity assay (Cytotoxicity Detection Kit; Roche Applied Science, Mannheim, Germany) was performed for BALF to determine lung injury. LDH activity was measured using a colorimetric assay according to the manufacturer’s instructions.

### Histopathology and tissue staining

Mouse left lungs were infused with 4% buffered paraformaldehyde, embedded in paraffin, sectioned at 4 μm thickness, and stained with hematoxylin and eosin to visualize morphology and to assess the extent of LPS-induced injury according to lung injury score as previously described^58^. The score was measured on 20 high-power fields (×400 magnification) of lung sections for each group. Moreover, immunohistochemical staining with primary antibodies against kallistatin, cleaved caspase-3, and Fas was performed. For detecting apoptosis, terminal deoxynucleotidyl transferase (TdT)-mediated dUTP–biotin nick-end labeling (TUNEL) staining DNA fragmentation assay kit was used according to the manufacturer’s instructions (Roche, Indianapolis, IN, USA). The number of positively stained cells per high-power field was counted on 3 different and randomly selected areas per specimen.

### Cell culture

Human lung epithelial A549 cell line was chosen for our *in vitro* study as it is commonly used in lung epithelial studies with LPS-induced inflammation and apoptosis^31^. Cells were grown on plastic dishes in Dulbecco’s modified Eagle’s medium DMEM (Gibco-BRL, Grand Island, NY, USA) containing L-glutamine and 15 mM HEPES, supplemented with 10% heat-inactivated FBS (Gibco-BRL), 100 units of penicillin and 100 μg/ml streptomycin, and maintained at 37 °C in 5% CO_2_.

### Apoptosis assay and caspase analysis

A549 cells were treated with LPS (100 μg/ml) to induce apoptosis. After drug treatment, cells were fixed with ice-cold 70% ethanol in PBS for propidium iodide (PI) (Sigma-Aldrich) staining and then were analyzed using flow cytometry (FACScan; BD Biosciences) as previously described^29^. Activation of caspase-3 and -8 was detected by western blotting and commercially available caspase-3 activity assay kits (BD Bioscience) and CaspaTag caspase-8 *in situ* assay kit (Merck Millipore, Germany) according to the manufacturer’s instructions.

### Immunostaining and flow cytometric analysis

For measurement of Fas levels, A549 cells were incubated in 10% FBS-DMEM. After drug treatment, cells were incubated with mouse anti-Fas antibody for 1 h at 4 °C. After a washing with PBS, cells were incubated with a FITC-conjugated goat anti-mouse IgG (Calbiochem, La Jolla, CA, USA) for a further 30 min on ice while protected from light. Cells were washed twice with PBS and detected in the FL-1 channel (515–545 nm) using FACSCalibur (BD Biosciences).

### Intracellular ROS measurement

Intracellular levels of ROS were determined by flow cytometry using 5-(and-6)- chloromethyl-2′,7′-dichlorodihydrofluorescein diacetate (CM-H_2_DCFDA; Invitrogen Life Technologies, Carlsbad, CA, USA) as fluorescent probes. Briefly, cells were incubated with the probes (10 μM) for 30 min in the dark. Then cells were washed, resuspended in PBS, and detected for fluorescence intensity using FACSCalibur with the FL-1 channel (515–545 nm).

### Kallistain and cytokine ELISA assays

Levels of kallistatin and cytokines/chemokines were measured by commercially available ELISA assay kits (R&D systems) according to the manufacturer’s instructions.

### Luciferase reporter assay

For the luciferase reporter assay, the cells were co-transfected with the NF-κB promoter-driven luciferase reporter (0.2 μg) and 0.01 μg of *Renilla* luciferase-expressing plasmid (pRL-TK; Promega, Madison, WI, USA) using the GeneJammer transfection reagent (Stratagene, La Jolla, CA, USA). Twenty-four h after the transfection, the cells were treated with LPS for 24 h with or without a 1-h pretreatment of kallistatin (0.5 μM) and NAC (10 mM). Cells were then lysed and harvested for luciferase and *Renilla* measurement using a luciferase assay system (Dual-Glo; Promega). For each lysate, the firefly luciferase activity was normalized to the *Renilla* luciferase activity to assess transfection efficiencies.

### Western blot analysis

For detecting the levels of kallistatin, Fas, FasL, TLR4, phospho-IκBα, caspase-3 and -8, and β-actin in lung tissues and A549 cells, immunoblotting was performed as previously described^29^. The densitometry of blots was quantified with Image J software.

### Statistical analysis

Data were expressed as means ± standard deviation (SD) or means ± standard error of the mean (SEM) if indicated. For human data, the Shapiro-Wilk test was used to assess the normality of distribution of investigated parameters. Because of skewed distribution, Spearman correlation-of-rank coefficient was used to analyze correlations between kallistatin and total protein in BALF, and between the BALF kallistatin/total protein ratio and PaCO_2_. To examine the impact of kallistatin in BALF on lung inflammation and the patients’ outcomes, we divided patients by quartile of kallistatin/total protein ratio. We eventually categorized patients into three groups by merging the highest two quartile due to they have similar patterns. For experimental data, variables with three or more groups were analyzed by one-way analysis of variance (ANOVA), followed by Bonferroni’s *post hoc* analysis. All tests were two-tailed and *P* < 0.05 was considered significant. The Kaplan–Meier survival curves were compared using the log-rank test. All data were analyzed by using a statistical software package (PASW for Windows, version 18.0; SPSS Inc, Chicago, IL, USA).

## Additional Information

**How to cite this article**: Lin, W.-C. *et al.* Kallistatin protects against sepsis-related acute lung injury via inhibiting inflammation and apoptosis. *Sci. Rep.* **5**, 12463; doi: 10.1038/srep12463 (2015).

## Supplementary Material

Supplementary Information
