# Supplementary material for: Kallistatin protects against sepsis-related acute lung injury via inhibiting inflammation and apoptosis
Source: Sci Rep. 2015 Jul 22;5:12463. doi: 10.1038/srep12463 (PMC4510498; doi:10.1038/srep12463)
Supplement: Supplementary Information [file srep12463-s1.pdf]

SUPPORTING MATERIAL

Kallistatin protects against sepsis-related acute lung injury via inhibiting inflammation and apoptosis

Wei-Chieh Lin[1]*, Chang-Wen Chen[1], Yu-Wen Huang[1], Lee Chao[2], Julie Chao[2], Yee-Shin Lin[3,4] & Chiou-Feng Lin[5,6]*

[1]Medical Intensive Care Unit, Department of Internal Medicine, National Cheng Kung University Medical College and Hospital, Tainan, Taiwan.
[2]Department of Biochemistry and Molecular Biology, Medical University of South Carolina, Charleston, South Carolina, USA.
[3]Department of Microbiology and Immunology, National Cheng Kung University Medical College, Tainan, Taiwan.
[4]Center of Infectious Disease and Signaling Research, National Cheng Kung University, Tainan, Taiwan.
[5]Graduate Institute of Medical Sciences, College of Medicine, Taipei Medical University, Taipei, Taiwan.
[6]Department of Microbiology and Immunology, College of Medicine, Taipei Medical University, Taipei, Taiwan.

***Correspondence:** W.-C. L., Medical Intensive Care Unit, Department of Internal Medicine, National Cheng Kung University Medical College and Hospital, 138 Sheng Li Road, Tainan 70101, Taiwan, e-mail: wclin@mail.ncku.edu.tw and C.-F. L., Department of Microbiology and Immunology, Taipei Medical University, Taipei 110, Taiwan, e-mail: cflin2014@tmu.edu.com

Results:

Supplementary Table S1

Table 1 | Characteristics of patients with sepsis-induced ARDS

Characteristics	Patients (n = 38)
Age, year	66.2 ± 15.7
Gender, male, n (%)	26 (68.4%)
Pneumonia/extrapulmonary sepsis (n)	27/11
APACHE II score, points	27.9 ± 7.6
SOFA score, points	11.1 ± 3.8
LIS score, points	2.7 ± 0.6
PaO_2/FiO_2	98.2 (43.4 - 432.2)
Hospital mortality, n (%)	17 (44.7%)

Data are presented as mean ± SD or median (range) for numerical variables or as number (%) for categorical variables.

ARDS: acute respiratory distress syndrome; APACHE: acute physiology and chronic health evaluation; SOFA: sequential organ failure assessment; LIS: lung injury score; PaO_2/FiO_2: the ratio of partial pressure of oxygen in arterial blood to fraction of inspired oxygen

Supplementary Figure S1

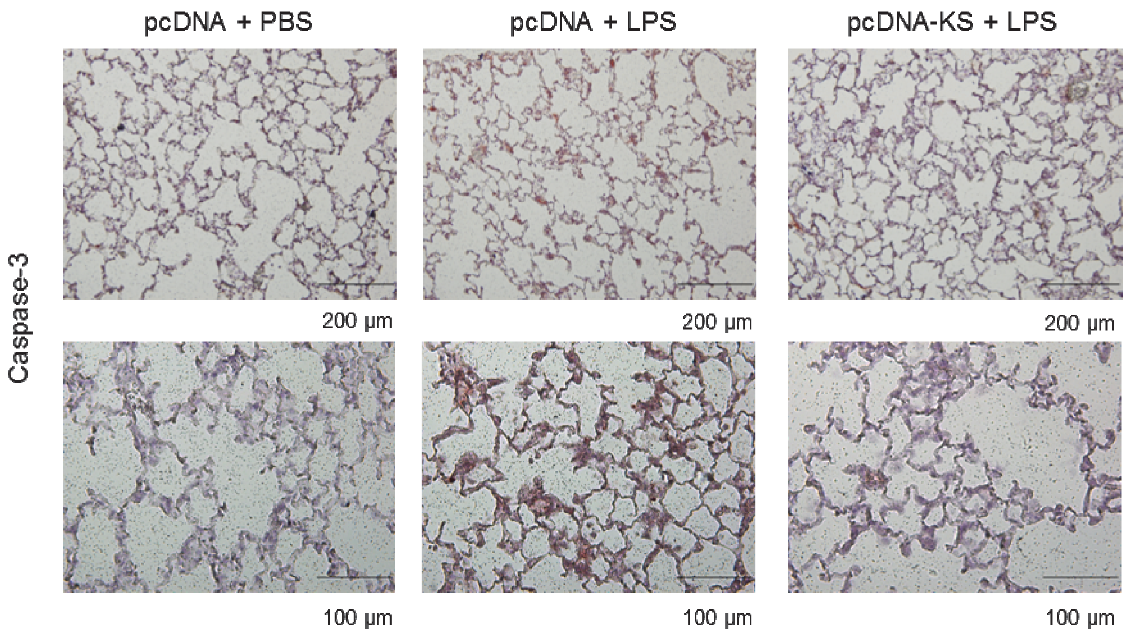

Figure S1 | Kallistatin gene transfer suppresses LPS-induced activation of caspase-3 in lung epithelial cells. Mice were intranasally treated with plasmid DNA encoding human kallistatin (pcDNA-KS) or control plasmid DNA (pcDNA). After 16 h, the mice were exposed to either LPS (50 µl, 100 mg/ml) or PBS for 24 h. Lung sections were stained for cleaved caspase-3. Results are representative of three independent experiments. *Brown* staining indicates cleaved caspase-3. Scale bars, 200 µm (top panel), 100 µm (bottom panel).

Supplementary Figure S2

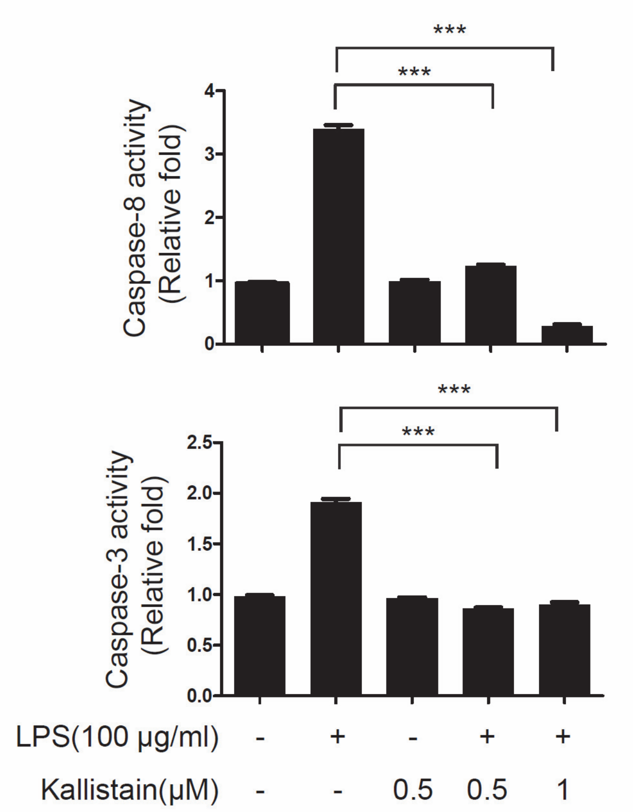

Figure S2 │ Kallistatin treatment reduces LPS-induced elevation of caspase-8 and -3 activity in A549 cells. A549 cells were pretreated with recombinant human kallistatin at indicated doses for 1 h and then exposed to LPS (100 μg/ml) for another 48 h. Data are shown as the mean ± SEM and are representative of two separate experiments. ***$P < 0.001$. Data were analyzed by one-way ANOVA with Bonferroni's *post hoc* test.

Supplementary Figure S3

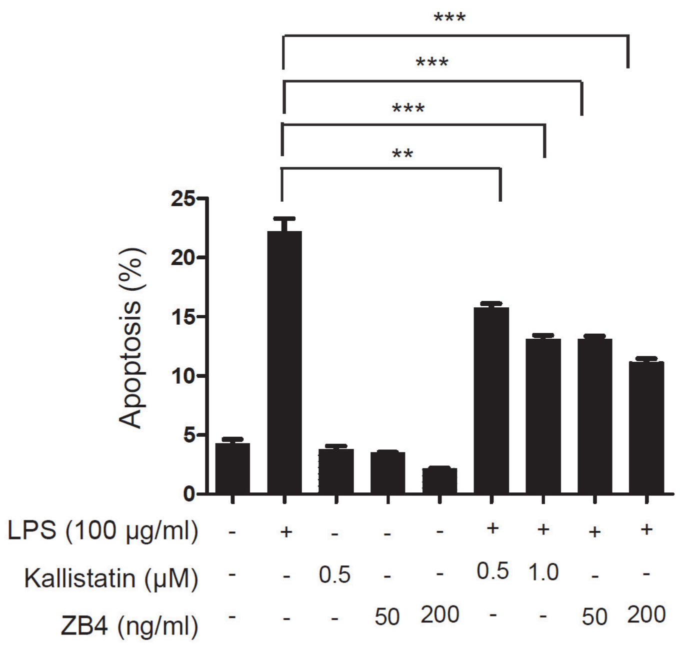

Figure S3 │ Kallistatin or Fas-blocking antibody ZB4 treatment attenuates LPS-induced apoptosis in A549 cells. Cells were pretreated with or without recombinant human kallistatin or ZB4 at indicated doses for 1 h and then exposed to LPS (100 μg/ml) for 48 h. Data are shown as the mean ± SEM and are representative of three independent experiments. $^{**}P < 0.01$ and $^{***}P < 0.001$. Data were analyzed by one-way ANOVA with Bonferroni's *post hoc* test.

Supplementary Figure S4

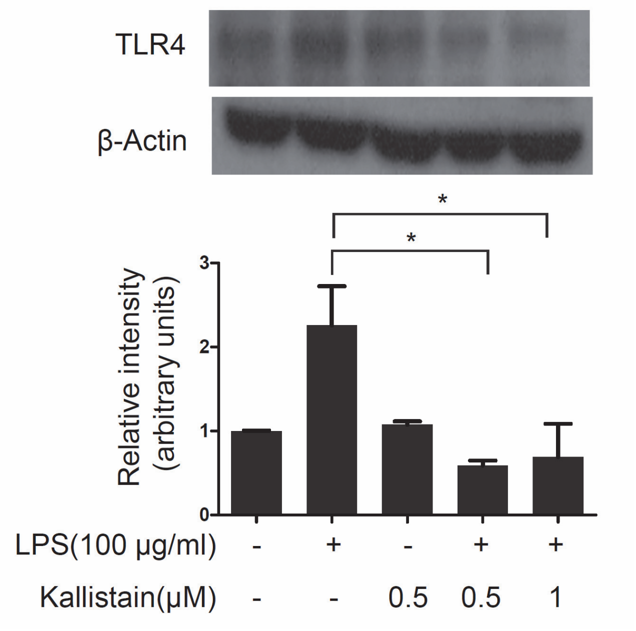

Figure S4 │ Kallistatin treatment inhibits LPS-induced TLR4 expression. A549 cells were exposed to LPS (100 μg/ml) or pretreated with recombinant human kallistatin at indicated doses for 1 h and then exposed to LPS for another 24 h. Western blot analysis was used to determine the expression of TLR4; β-actin was used as an internal control. Results are representative of three independent experiments. Histogram shows the relative band intensity of western blot from three independent experiments. Data are shown as the mean ± SEM and were analyzed by one-way ANOVA with Bonferroni's *post hoc* test. $^*P < 0.01$.
